# An assessment of national surveillance systems for malaria elimination in the Asia Pacific

**DOI:** 10.1186/s12936-017-1774-3

**Published:** 2017-03-21

**Authors:** Chris Erwin G. Mercado, Nattwut Ekapirat, Arjen M. Dondorp, Richard J. Maude

**Affiliations:** 1 Mahidol Oxford Tropical Medicine Research Unit, Faculty of Tropical Medicine, Mahidol University, Bangkok, Thailand; 2 Department of Tropical Hygiene, Faculty of Tropical Medicine, Mahidol University, Bangkok, Thailand; 3 Centre for Tropical Medicine and Global Health, Nuffield Department of Medicine, University of Oxford, Oxford, UK; 4 Harvard T.H. Chan School of Public Health, Harvard University, Boston, USA

**Keywords:** Malaria elimination, Surveillance, National Malaria Control Programme, Epidemiology, Asia Pacific

## Abstract

**Background:**

Heads of Government from Asia and the Pacific have committed to a malaria-free region by 2030. In 2015, the total number of confirmed cases reported to the World Health Organization by 22 Asia Pacific countries was 2,461,025. However, this was likely a gross underestimate due in part to incidence data not being available from the wide variety of known sources. There is a recognized need for an accurate picture of malaria over time and space to support the goal of elimination. A survey was conducted to gain a deeper understanding of the collection of malaria incidence data for surveillance by National Malaria Control Programmes in 22 countries identified by the Asia Pacific Leaders Malaria Alliance.

**Methods:**

In 2015–2016, a short questionnaire on malaria surveillance was distributed to 22 country National Malaria Control Programmes (NMCP) in the Asia Pacific. It collected country-specific information about the extent of inclusion of the range of possible sources of malaria incidence data and the role of the private sector in malaria treatment. The findings were used to produce recommendations for the regional heads of government on improving malaria surveillance to inform regional efforts towards malaria elimination.

**Results:**

A survey response was received from all 22 target countries. Most of the malaria incidence data collected by NMCPs originated from government health facilities, while many did not collect comprehensive data from mobile and migrant populations, the private sector or the military. All data from village health workers were included by 10/20 countries and some by 5/20. Other sources of data included by some countries were plantations, police and other security forces, sentinel surveillance sites, research or academic institutions, private laboratories and other government ministries. Malaria was treated in private health facilities in 19/21 countries, while anti-malarials were available in private pharmacies in 16/21 and private shops in 6/21. Most countries use primarily paper-based reporting.

**Conclusions:**

Most collected malaria incidence data in the Asia Pacific is from government health facilities while data from a wide variety of other known sources are often not included in national surveillance databases. In particular, there needs to be a concerted regional effort to support inclusion of data on mobile and migrant populations and the private sector. There should also be an emphasis on electronic reporting and data harmonization across organizations. This will provide a more accurate and up to date picture of the true burden and distribution of malaria and will be of great assistance in helping realize the goal of malaria elimination in the Asia Pacific by 2030.

## Background

Malaria remains a major cause of death and illness with an estimated 2.1 billion people in the Asia Pacific at risk of infection [1]. With the growing threat of anti-malarial drug resistance and the urgent need to contain its spread in the Greater Mekong Subregion (GMS) [2–5], malaria is a major regional public health concern. Not recognizing national borders, control and elimination will require coordination between countries and across sectors to minimize the further loss of lives and valuable resources.

At the 2013 East Asia Summit (EAS), the Asia Pacific Leaders Malaria Alliance (APLMA) was established to accelerate progress towards a reduction in malaria cases and deaths. In 2014 at the ninth EAS, the APLMA Co-Chairs (the Prime Ministers of Viet Nam and Australia) tabled a recommendation for the Asia Pacific region to become free of malaria by 2030. The EAS Heads of Government agreed to the goal, and tasked APLMA Co-Chairs to present a plan to reach malaria elimination through a “Leaders Malaria Elimination Roadmap” [6]. This roadmap was presented to Heads of Government during the 10th EAS Meeting in 2015 [7]. With support from the Australian government, APLMA worked with the Mahidol Oxford Tropical Medicine Research Unit (MORU) to build an evidence base that will be used to inform decisions on malaria elimination. MORU developed a report on the status of malaria elimination in the Asia Pacific and collected data on malaria surveillance from each National Malaria Control Programme (NMCP) in the Asia Pacific.

**Fig. 1:**
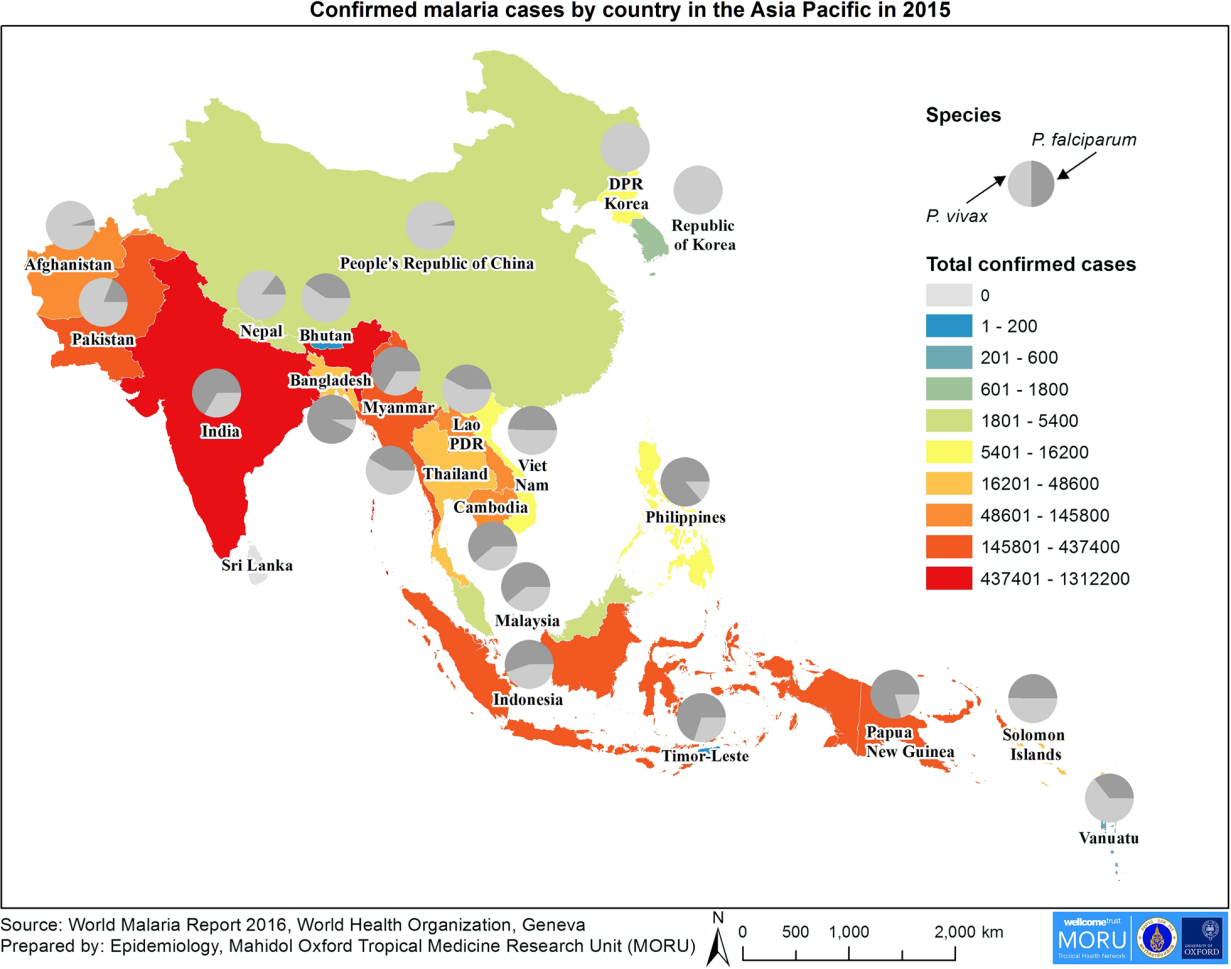
Confirmed malaria cases in 22 Asia Pacific countries in 2015 [8]

For malaria elimination to succeed, it is essential to have an accurate picture of malaria incidence over time and space. In 2015, the total number of confirmed cases reported to the World Health Organization (WHO) by 22 Asia Pacific countries identified by APLMA was 2,461,025 (Fig. 1) [8]. However, in many countries, available information on malaria incidence is incomplete. Most NMCPs routinely collect malaria incidence data for surveillance predominantly from government health facilities and this is the main dataset that is reported to WHO. The completeness of these data and extent to which data from hospitals and community health workers are included is thought to vary greatly. In many countries, a range of other organizations including non-governmental organizations (NGOs), the private sector and the military have a major role in providing malaria care but do not contribute data to the NMCP malaria surveillance system. Even where some of this information is available, it often does not reach the NMCP quickly enough for timely response planning. This incomplete reporting of malaria incidence can result in a very inaccurate picture of the distribution of malaria and a gross underestimate of the disease burden for the NMCP. However, the extent to which malaria incidence data are collected from these various sources by each country is not well described.

Incomplete reporting of malaria incidence greatly impedes allocation of appropriate resources within governments and impairs efficient targeting of malaria control interventions. It also constrains access to external funding where disease burdens are underestimated. A complete and accurate picture of malaria incidence is essential to show reliable evidence that a country is moving towards elimination [9, 10]. Additionally, harmonized sharing of reliable and complete data between countries would facilitate direct comparability of datasets across international borders. Ongoing efforts to increase this harmonization will help underpin regional conversations about malaria elimination and will greatly improve coordination between NMCPs.

This study assessed the current sources of malaria surveillance data collected by NMCPs and the role of the private sector in malaria treatment in the Asia Pacific region. At the time of this survey, target countries had either ongoing local malaria transmission or were working towards certification for malaria elimination [11, 12].

## Methods

**Fig. 2:**
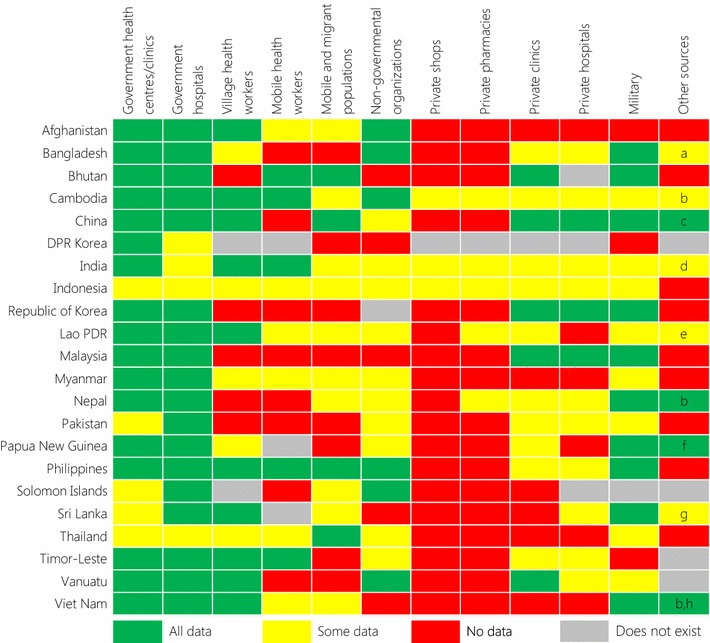
Questionnaire responses on sources of malaria incidence data collected by each NMCP. Additional sources of data were identified in nine countries and were labeled as follows: *a* tea estates; *b* police; *c* sentinel surveillance sites; *d* railways, Central Government Health Scheme, Employees’ State Insurance and Public Sector Undertakings; *e* police provincial hospitals, provincial anti-malaria stations; *f* Institute of Medical Research sites; *g* private laboratories, university parasitology departments; *h* Border guards, Ministries of Agriculture and Rural Development, Transportation, Industry and Trade. ‘All data’/‘Some data’/‘No data’ = the extent to which data from that source are collected by the NMCP. ‘Does not exist’ indicates that the source does not exist in that country

**Fig. 3:**
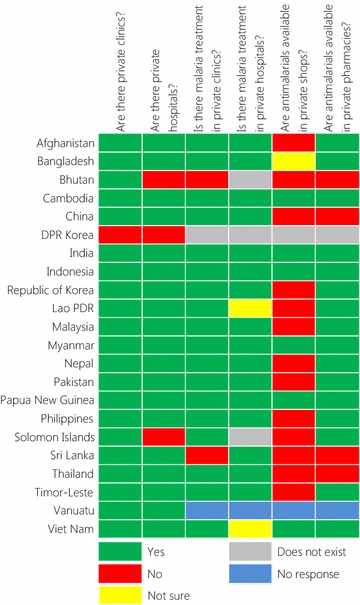
Questionnaire responses about private sector treatment of malaria in each country. The questions asked are shown at the top. ‘Not sure’ the respondent did not know the answer, ‘Does not exist’ that entity does not exist in that country, ‘No response’ no response was provided to that question

A short self-administered questionnaire was developed to collect information from each country about the system for collection of malaria incidence data and treatment in the private sector. The survey was developed in consultation with staff from selected NMCPs and regional policymakers. Following iteration incorporating their feedback, it was designed to be as short and clear as possible to maximize the likelihood of receiving responses while maintaining clarity of the information collected. The final questions were in three sections. Section (a) what sources of malaria incidence data are collected by the NMCP in (country) … ? A row was provided for each category listed in Fig. 2 with choices of “all”, “some” or “none” for the extent to which data from each source was included in the national surveillance database plus a space to add free text comments or clarifications for each category. Space was also provided for countries to add any additional sources not already listed. Section (b) details were requested of the system for collecting and collating incidence data in that country. Section (c) a set of questions to determine the role of the private sector in malaria treatment, as shown in Fig. 3.

In 2015 to 2016, the survey was conducted with the assistance of APLMA among 22 target countries in the Asia Pacific: Afghanistan, Bangladesh, Bhutan, Cambodia, People’s Republic of China (hereafter referred to as China), Democratic People’s Republic of Korea (DPR Korea), India, Indonesia, Republic of Korea, Lao People’s Democratic Republic (Lao PDR), Malaysia, Myanmar, Nepal, Pakistan, Papua New Guinea, Philippines, Solomon Islands, Sri Lanka, Thailand, Timor-Leste, Vanuatu and Viet Nam. The data collection tool was either administered in person or sent through email to key persons in the NMCP of each country. Follow-up was done by telephone and email to clarify unclear or incomplete responses. Survey data were encoded and stored in a secured database (Microsoft Access 2013). Summary figures were produced to facilitate effective communication of findings to policymakers and the APLMA member country Heads of Government.

## Results

### Description of survey participants

All 22 country NMCPs completed the survey by May 2016. Of these, 14 respondents (64%) identified themselves as the NMCP director or manager, five (23%) were staff members from the NMCP and three (14%) others worked in close collaboration with the NMCP. Survey results on the different sources of malaria surveillance data as collected by NMCPs are shown in Fig. 2.

### Sources of malaria surveillance data according to NMCPs

#### Government sources

Twenty-two countries reported that they collect malaria incidence data from government health facilities. Seventeen collected all information from health centres/clinics while five only collected some. In hospitals, 18 collected all information while four only collected some. In 21 countries that diagnose malaria in the military (not the Solomon Islands), only 10 collected all data while eight others collecting some data. The police and other security forces were also identified as sources of malaria incidence data in Cambodia, Nepal and Viet Nam.

#### Village health workers, mobile health workers, mobile and migrant populations

Out of 20 countries with village health workers (not DPR Korea and Solomon Islands), all malaria incidence data were included by 10 whereas five only collected some data. In the 19 countries with mobile heath workers (not DPR Korea, Papua New Guinea and Sri Lanka), five collected all data while six only collected some data. Only four NMCPs collected all data and 10 only collected some from mobile and migrant populations.

#### Non-governmental organizations

NGOs were reported to provide some malaria care in 21 countries, except for the Republic of Korea. Of those countries, six collected all incidence data from NGOs while 10 collected some data.

#### Private sector

Information collected on the role of the private sector in malaria care is summarized in Fig. 3. The private sector for health was reported to exist in all countries except in DPR Korea. Information on private sector malaria treatment or availability of anti-malarials were not available for Vanuatu. It was reported that malaria was being treated in the private sector in 19 of 20 (95%) countries (with the exceptions of Bhutan and Sri Lanka). All these 19 countries reported that malaria treatment occurred in private health facilities (clinics or hospitals). All data from private clinics were collected in five countries while some data were included in 10. Three countries reported that they do not have private hospitals (Bhutan, DPR Korea and Solomon Islands). Of those countries with private hospitals, 16 reported that they treat malaria, while two were uncertain. Three countries collected all, and 10 collected some malaria incidence data from private hospitals.

Anti-malarials were reported as being available in private pharmacies in 16 countries and private shops in six of these same countries. Of these, shops or pharmacies were reported as a source of some incidence data in five (31%) countries. Only four countries in the region stated that anti-malarials were not available in private shops or pharmacies (Bhutan, China, Sri Lanka and Thailand).

#### Other sources

Other sources of malaria incidence data were cited by some countries in the Asia Pacific, with varying extents of data collection. These included data from plantations, sentinel surveillance sites, research or academic institutions, private laboratories and government agencies outside the Ministry of Health.

### Situation in the Greater Mekong Subregion

Five of six countries in the GMS (except Thailand) collected all information on malaria incidence from government health facilities. In addition, village health workers, mobile and migrant populations and the military also contributed to the national surveillance datasets of all GMS member countries. All countries in the GMS reported treatment of malaria in the private sector. No information on malaria incidence was collected by NMCPs from the private sector in Myanmar, Thailand or Viet Nam.

### System for collecting and collating malaria incidence data in the Asia Pacific

Fourteen of 17 (82%) countries reported that they use paper-based collection of incidence data at the level of individual health facilities and/or healthcare providers. These data were then aggregated and entered into an electronic database. The administrative level at which this electronic data entry occurred varied widely between countries. Seven of these 15 (47%) countries reported that they have in addition, or were in the process of rolling out, an electronic reporting system (short text messaging, smartphone or web-based) at facility level. Three of 17 (18%) countries reported already using primarily electronic reporting. Four countries did not specify about paper-based versus electronic data collection and one country provided no reply for this section of the survey.

## Discussion

This survey demonstrates the wide range of potential sources of malaria surveillance data across the Asia Pacific region. Being across a wide range of organizations, it can be very challenging for an NMCP to collect data from all of these sources. In all countries, the main source of malaria incidence data as collected by NMCPs was from government health facilities from which data were complete in two-thirds of countries in this study. Half of the countries collected complete information from village health workers, whose contribution has been considered crucial in achieving malaria elimination [13–15], although they were present in all but two. Collection of data from mobile and migrant populations, NGOs, the private sector and the military—all potentially important sources of malaria data in many of these countries—was much less comprehensive. Much malaria treatment occurred in the private sector (clinics, hospitals, pharmacies and shops) in most countries but few collected data from these sources. Engaging the private sector is one of the major challenges for malaria elimination in many countries [16, 17], although there have been some notable successes [18]. This survey also documented the existence of additional but potentially important sources of malaria surveillance data in nine countries including other government ministries and the police. Overall, these findings suggest malaria incidence data in countries across the Asia Pacific region is missing information from a wide range of potential sources. This study did not attempt to quantify the possible magnitude of the incidence information that could be collected from these sources, although it has been suggested that in some countries, for example Pakistan, the majority of cases may be missed [16]. Attempts have been made to try to estimate the overall number of malaria cases in each country, most notably by the WHO for the annual World Malaria Report [8]. However, such estimates have a wide range of uncertainty due to the limitations of the available data. This lack of a complete picture of malaria burden could hinder current progress made towards national and regional malaria elimination goals [3, 6, 19]. The WHO characterized a malaria surveillance system as being effective if it could enable programme managers to identify the areas or population groups mostly affected by malaria, identify trends in cases and deaths that require additional interventions and assess the impact of control measures [9]. The success of planning for control and elimination relies heavily on having an accurate picture of malaria incidence, mainly through surveillance [20].

In the light of these findings, the authors propose two general recommendations. First, a regional effort is warranted to include additional sources of malaria case data in the national surveillance database for each country, in particular from village health workers, mobile and migrant populations, the private sector, NGOs and the military. This should include facilitating links for the NMCPs to engage with a much wider community of data collectors. Second, there should be continued support in countries to improve the completeness of malaria surveillance data collected from existing sources. Courses of action would necessitate steady leadership, information infrastructure and funding support, both within and between countries in the region [21–23]. In addition, there is a need to build an international network of collaborators on assembly of malaria data from the various sources and to facilitate sharing of solutions and best practices to help move the malaria information base closer to comprehensive coverage.

Most countries collected incidence data at facility level using paper forms and aggregated data for entry into an electronic database at different administrative levels that varied widely between countries. Many countries also had, or were in the process of rolling out, electronic data collection systems (short text messaging, smartphone or web-based). Although often expensive to implement, electronic data collection systems have the advantage that data can be collected into a central Health Information System more rapidly thus potentially providing a more up to date picture for the NMCP and increasing the efficiency of elimination efforts [24, 25]. A third recommendation from this study is that wherever possible, collection of incidence data from all sources should be done electronically at the level of the individual facility or healthcare provider into a centralized system accessed by the NMCP. Collation of data from multiple sources within a country is another major potential bottleneck with different organizations using their own reporting formats and standalone database systems. Electronic data collection and collation should thus ideally include harmonization of data reporting formats and use of a single electronic data collection tool across multiple organizations.

For malaria elimination to succeed, it is important to have an up to date regional picture of malaria burden. This is particularly important in areas with cross-border movement of people with malaria where elimination efforts should be coordinated between countries and detailed information from either side of borders is needed. In Fig. 1, the authors created a map showing a summary of confirmed malaria cases by country. This was produced using publicly available data. Producing maps using subnational level data from multiple countries is currently far more challenging with inaccurate or unavailable geographic information, out of date population estimates and no common platform for sharing and harmonization of disease data between countries at subnational level. Various efforts are underway to remedy this.

This study had some limitations. In order to achieve a high response rate from multiple busy NMCPs, the survey was necessarily brief and simplistic. It was in no way a comprehensive assessment of surveillance systems, focusing instead on three priority areas. The survey responses were selected from pre-specified options that were qualitative in nature. To elicit more context, respondents were given the chance to write their remarks after each close-ended item, although not all of them did so. Country NMCPs were also asked to describe the system for how they collect and collate malaria incidence data, including the role of the private sector in treatment. Moreover, the profile of respondents varied in terms of experience and function in the NMCP. In an attempt to gain insights from the most qualified person, survey forms were initially sent out to NMCP directors or managers in each country. In countries where this was not possible, this was delegated and other qualified staff or collaborators were able to provide responses. The responses were necessarily categorical due to a lack of quantitative information on the relative contribution of the various sources. It is possible that there are other potential sources of malaria incidence data that were not identified by the survey respondents. In addition, it is likely that within individual categories there will be sources about which the NMCP is unaware, for example specific private sector providers like mining companies [26] and plantations [27].

Efforts to improve surveillance are ongoing and some of the responses here may no longer apply. Despite the limitations, the study found some important patterns in the way malaria incidence data are collected in multiple countries. This facilitates the identification of gaps in malaria surveillance and promotes regional planning of strategies for malaria elimination and control.

## Conclusions

Most collected malaria incidence data in the Asia Pacific is from government health facilities while data from a wide variety of other known sources are not included. In particular, there needs to be a concerted regional effort to support inclusion of data on mobile and migrant populations and the private sector. There should also be an emphasis on electronic reporting and data harmonization across organizations. This will provide a much more accurate and up to date picture of the true burden and distribution of malaria and will be of great assistance in helping realize the goal of malaria elimination in the Asia Pacific by 2030.

## Abbreviations

APLMA: Asia Pacific Leaders Malaria Alliance
DPR: Democratic People’s Republic of Korea
EAS: East Asia Summit
GMS: Greater Mekong Subregion (Cambodia, Yunnan Province in China, Lao People’s Democratic Republic, Myanmar, Thailand and Viet Nam)
MORU: Mahidol Oxford Tropical Medicine Research Unit
NGO: non-governmental organization
NMCP: National Malaria Control Programme
PDR: Lao People’s Democratic Republic
WHO: World Health Organization
